# Characteristics and Expression of circ_003628 and Its Promoted Effect on Proliferation and Differentiation of Skeletal Muscle Satellite Cells in Goats

**DOI:** 10.3390/ani12192524

**Published:** 2022-09-21

**Authors:** Huimin Zhen, Jiyuan Shen, Jiqing Wang, Yuzhu Luo, Jiang Hu, Xiu Liu, Shaobin Li, Zhiyun Hao, Mingna Li, Bingang Shi, Yuanhua Gu

**Affiliations:** Gansu Key Laboratory of Herbivorous Animal Biotechnology, College of Animal Science and Technology, Gansu Agricultural University, Lanzhou 730070, China

**Keywords:** circ_003628, skeletal muscle satellite cells, proliferation, differentiation, expression, goat

## Abstract

**Simple Summary:**

Circular RNAs (circRNAs) are new regulators of the development of skeletal muscle in mammals. Herein, circ_003628 in *Longissimus dorsi* muscle tissue of goats, previously found by RNA-seq, was selected to construct an expression profile in different caprine tissues, and investigate the effect on proliferation and differentiation of caprine skeletal muscle satellite cells (SMSCs), using RT-qPCR, EdU, CCK-8 and immunofluorescence assays. The results showed that circ_003628 had the highest expression level both in the *longissimus dorsi* muscle among nine caprine tissues collected, and on day 6 after differentiation during SMSCs differentiation periods. The interfering of circ_003628 inhibited the viability, proliferation, and differentiation of goat SMSCs.

**Abstract:**

In our previous a study, circ_003628 was one of the most highly expressed circular RNAs (circRNAs) in the *Longissimus dorsi* muscle of goats found by RNA-seq, suggesting that the circRNA may be important for caprine muscle growth and development. However, there have been no reports describing the molecular mechanisms by which circ_003628 regulates the activities of goat skeletal muscle satellite cells (SMSCs). In this study, reverse transcriptase-PCR (RT-PCR) and DNA sequencing were used to validate the authenticity of circ_003628, and its characteristics, expression profile and effect on goat SMSCs were also studied using real-time quantitative-PCR (RT-qPCR), EdU, CCK-8 and immunofluorescence assays. Circ_003628 is partially originated from 13 exons, 12 introns and 3′-untranslated regions (UTR) of caprine Myosin Heavy Chain 1 (*MYH1*), and 25 exons and 5′ UTR of Myosin Heavy Chain 4 (*MYH4*), as well as intergenic sequences between the two genes. A total of 77.07% of circ_003628 were located in the nuclei of goat SMSCs, while 22.93% were expressed in the cytoplasm. The circRNAs were only expressed in *triceps brachii*, *quadriceps femoris* and *longissimus dorsi* muscle tissues in nine caprine tissues investigated, with the highest expression level in *longissimus dorsi* muscle. The expression level of circ_003628 gradually increased during differentiation periods of goat SMSCs and reached the maximum on day 6 after differentiation. The small interfering RNA of circ_003628 (named si-circ_003628) inhibited the viability and proliferation of goat SMSCs, and also decreased the expression of four cell proliferation marker genes: paired box 7 (*Pax7*), cyclin-dependent kinase 2 (*CDK2*), *CDK4* and *CyclinD1* in goat SMSCs. Transfection of si-circ_003628 significantly decreased the area of MyHC-labeled myotubes of goat SMSCs, as well as the expression levels of three differentiation marker genes: myosin heavy chain (*MyHC*), myogenin (*MyoG*), and myocyte enhancer factor 2C (*MEF2C*). These results suggest that circ_003628 promotes the viability, proliferation, and differentiation of goat SMSCs, and they also provide an improved understanding of the roles of circ_003628 in skeletal muscle growth and development in goats.

## 1. Introduction

Circular RNAs (circRNAs) were first discovered in plant viroids in 1976 [1]. They are a class of non-coding RNAs and originated from back-splicing of their parent genes [2]. As a result of the absence of 5′-cap and 3′-poly (A) tail structures, circRNAs are not easily degraded by RNA exonuclease. In this context, circRNAs are more stable than linear RNAs [3,4,5]. The circRNAs can be classified into six types according to their sequences derived from the parent genes: one_exon, annot_exon, intron_exon, intronic, intergenic and antisense circRNAs [6].

It has been proved that circRNAs have definite biological functions that affect phenotypic traits, though the functions of most circRNAs have not been clearly discovered. Some circRNAs can increase the expression level of mRNAs by acting as microRNA (miRNA) sponges [7,8]. Additionally, exon-intron circRNAs, mainly located in nuclei, are involved in the expression regulation of their parent genes [9]. Finally, some circRNAs can also be translated into functional proteins [10], or play important biological roles by interacting with RNA binding proteins [11,12].

With the development of RNA-Seq and bioinformatics technologies, thousands of unique circRNAs have been annotated in skeletal muscle tissues in chickens [13], cattle [14], goats [15], pigs [16], and sheep [17]. For example, Lei et al. [18] identified 5755 differentially expressed circRNAs in breast muscle tissue of Shouguang chickens between embryonic periods and postnatal periods. A total of 214 differentially expressed circRNAs were found in the *longissimus dorsi* muscle between two goat breeds with different meat yield and quality, and their parent genes were enriched in connective tissue development, Rap1, cGMP-PKG, cAMP and Ras signaling pathway [15]. These studies confirmed differential expression of circRNAs in skeletal muscle tissues collected from different development periods or different breeds with phenotypic differences in meat production performance. However, the function of these circRNAs were investigated more by analyzing the function enrichment of their parent genes using Gene Ontology (GO) and the Kyoto Encyclopedia of Genes and Genomes (KEGG) analysis database. The functions of most circRNAs in muscle tissue identified by RNA-Seq have not been further verified in vivo or in vitro. 

Up to now, research into the effect of individual circRNA on caprine skeletal muscle activities is limited. CircUSP13 promoted differentiation of goat primary myoblasts by increasing *IGF1* expression [19]. CircRNA CDR1as increased the expression of *ANGPT1* by acting as a sponge of miR-27a-3p, ultimately promoting myoblast differentiation in goat skeletal muscle satellite cells (SMSCs) [20].

In our previous research, circ_003628 was found to be highly expressed with a Reads Per Million mapped reads (RPM; a normalized expression level measure of the circRNA) value of 3228 in the *longissimus dorsi* muscle of Ziwuling black goats [15]. This suggests that circ_003628 may be important for muscle growth and development. However, the characteristics of circ_003628 in expression and its effect on goat SMSCs are still unknown. Accordingly, in this study, we check the presence of circ_003628 and analyze its features. Moreover, we investigate the expression levels of circ_003628 and its parent genes in caprine nine tissues and SMSCs during different differentiation periods, and the effect of circ_003628 on viability, proliferation and differentiation of goat SMSCs. The results lay a theoretical foundation for elucidating the biological role of circ_003628 in caprine muscle development.

## 2. Materials and Methods

### 2.1. Sample Collection

The Animal Experiment Ethics Committee of Gansu Agricultural University approved our experiment animal studies with an approval number of GSAU-ETH-AST-2021-028.

Four healthy nine-month-old Ziwuling black goat rams were slaughtered, and nine tissue samples were collected from each goat, including samples of *longissimus dorsi* muscle, *quadriceps femoris* muscle, *triceps brachii* muscle, heart, liver, spleen, lung, kidney, and testis tissues. The samples were immediately frozen in liquid nitrogen and then stored at −80 °C. A part of *longissimus dorsi* muscle tissues were also collected for SMSC culture.

### 2.2. Isolation, Purification and Culture of SMSCs

SMSCs were isolated using the methods described by Ling et al. [21] and Sui et al. [22]. Briefly, after a quick washing step with sterile phosphate-buffered saline (PBS, Hyclone, Logan, UT, USA), and removing visible fascia, connective tissue and adipose tissue from *Longissimus dorsi* muscle tissues, the muscle samples were cut with scissors and digested with 0.1% collagenase I (Solarbio, Beijing, China) and 0.25% trypsin (Hyclone, Logan, UT, USA) to release cells. The cell suspension was filtered and the SMSCs were correspondingly collected. After being washed three times with PBS, the SMSCs were centrifuged and then resuspended using DMEM/F12 medium (Hyclone, Logan, UT, USA) containing 20% fetal bovine serum (Invigentech, Carlsbad, CA, USA). Finally, the SMSCs were cultured at 37 °C with 5% CO_2_. 

Based on slower adhesion of SMSCs to plastic, a differential adhesion method described by Ling et al. [21] and Sui et al. [22] was used to remove other cell types (predominantly fibroblasts). After purification for three times, a rabbit anti-Paired Box 7 (*Pax7*) polyclonal antibody (Absin, Shanghai, China), which is a specific marker of SMSCs, was used to identify SMSCs, as suggested by Ling et al. [21] and Feng et al. [23]. The previous result of cell identification showed that isolated cells were SMSCs of high purity, indicating the cells could be used for the further investigation.

In general, purified SMSCs were cultured in growth medium (69% DMEM/F12 medium, 20% fetal bovine serum, 10% horse serum, and 1% penicillin/streptomycin) at 37 °C with 5% CO_2_, and then used for nuclei and cytoplasm isolation, differentiation and transfection of SMSCs.

### 2.3. The Authenticity Verification of circ_003628

The authenticity of circ_003628 was validated using the Reverse transcriptase-polymerase chain reaction (RT-PCR) and DNA sequencing. Divergent primers that contained head-to-tail splice junctions were designed for circ_003628 (Table 1), and RT-PCR amplification was performed. The amplified products were visualized using 1.5% agarose gel electrophoresis and subsequently sequenced. In order to check the presence of the back-splicing junction site of circ_003628, MEGA v7.0 was used to align sequences produced by DNA sequencing with sequences from RNA-Seq and Caprine Genome Assembly ARS1.

### 2.4. Cellular Localization of circ_003628

The nuclei and cytoplasm of SMSCs were isolated using Minute^TM^ Cytoplasmic and Nucleus Isolation Kit (Invent, Minneapolis, MN, USA). The RNA was extracted and then reverse transcribed to produce cDNA. Reverse transcriptase-quantitative-PCR (RT-qPCR) was performed for circ_003628. The caprine *U6* and *β-actin* were chosen as internal references for the normalization of expression level in nuclei and cytoplasm, respectively [24], with the primers for the amplification of the sequences listed in Table 1.

### 2.5. The Tissue Expression of circ_003628 and Its Parent Genes

Total RNA from nine caprine tissues and SMSCs was extracted using TRIzol reagent (Invitrogen, Carlsbad, CA, USA). The concentration and purity of the RNA was checked using a NanoDrop 8000 spectrophotometer (NanoDrop Technologies, Wilmington, NC, USA). The cDNA was synthesized with a HiScript III 1st Strand cDNA Synthesis Kit (Vazyme, Nanjing, China).

The RT-qPCR analysis was performed in triplicate with the 2 × SYBR Green qPCR Master Mix (Vazyme, Nanjing, China) on an Applied Biosystems QuantStudio 6 Flex Real-time PCR System (Thermo Fisher Scientific, Waltham, MA, USA). *GAPDH* was used as an internal control to normalize the expression of circ_003628 and its parent genes [19]. The 2^−^^ΔΔ^^ct^ method was used to calculate their relative expression levels. The information of RT-qPCR primers is listed in Table 1. 

### 2.6. Cell Transfection, CCK-8 and EdU Assays

The SMSCs were cultured in 96-well plates and 24-well plates using growth medium, respectively. The small interfering RNA of circ_003628 (named si-circ_003628) and its corresponding negative controls (named si-NC) were synthesized by Ribobio company (Ribobio, Guangzhou, China). When the density of SMSCs cultured in 96-well plates and 24-well plates for each well reached approximately 70 to 80%, si-circ_003628 and si-NC were, respectively, transfected into SMSCs using the INVI DNA and RNA Transfection Reagent™ (Invigentech, Carlsbad, CA, USA). For SMSCs cultured in 96-well plates, after transfection for 48 h, 10 μL of CCK-8 reagent (Vazyme, Nanjing, China) was added into each well, and then cultured for 2 h in a 37 °C incubator. The absorbance of SMSCs at 450 nm was measured with a microplate reader (Thermo Scientific, Waltham, MA, USA). 

For SMSCs cultured in 24-well plates, after transfection for 48 h, growth medium containing 20 μM EdU reagent (Beyotime, Shanghai, China) was added to each well, and the cell culture lasted 4 h. The IX73 (Olympus, Tokyo, Japan) microscope was used to observe the number of EdU-labeled positive SMSCs, and images were also randomly captured. The percentage of EdU-labeled positive SMSCs was calculated using ImageJ software (National Institutes of Health, Bethesda, Montgomery, MD, USA). Meanwhile, after transfection for 48 h, the total RNA from SMSCs was extracted and the expression levels of the following five proliferation marker genes, circ_003628 and their parent genes *MYH1* and *MYH4* were detected using RT-qPCR: paired box 7 (*Pax7*), cyclin-dependent kinase 2 (*CDK2*), *CDK4*, *CyclinD1* and *p27*. *β-actin* was used as an internal control to normalize the expression of the genes [25]. The primer information of the genes described above is shown in Table 1.

### 2.7. Induced Differentiation of SMSCs

When the density of SMSCs cultured in growth medium reached about 80%, the growth medium was replaced by differentiation medium (97% DMEM/F12 medium, 2% horse serum and 1% penicillin/streptomycin) to induce differentiation of SMSCs. On days 0, 2, 4, 6 and 8 after the initiation of differentiation, the total RNA from SMSCs was extracted for the expression detection of the following two myogenic differentiation marker genes: myogenic differentiation antigen (*MyoD*) and myocyte enhancer factor 2C (*MEF2C*), circ_003628 and its parent genes *MYH1* and *MYH4*.

When the density of SMSCs reached 80% to 90%, si-circ_003628 and si-NC were transfected into the SMSCs, using the method described above. After transfection for 24 h, the induction differentiation of SMSCs was initiated using the differentiation medium. On day 6 after differentiation, SMSCs were stained overnight at 4 °C using the primary antibody, monoclonal anti-MyHC antibody (R&D Systems, Minneapolis, MN, USA), and then washed 3 times with PBS (5 min each). Subsequently, the cells were incubated at room temperature for 2 h with corresponding secondary antibody, goat anti-mouse IgG (H+L) CY3-conjugated (Affinity, Melbourne, Australia), and then washed 4 times with PBS. Nuclei were visualized by Hoechst 33342 (Solarbio) stain and then observed using a IX73 microscope. Subsequently, the relative area of MyHC-labeled positive myotubes was counted using ImageJ. Meanwhile, the total RNA of SMSCs was extracted and the expression levels of the following myogenic differentiation marker genes, circ_003628 and their parent genes *MYH1* and *MYH4* were detected using RT-qPCR on day 6 of induction differentiation: myosin heavy chain (*MyHC*), myogenin (*MyoG*), *MEF2C* and transforming growth factor beta 1 (*TGFβ1*). *β-Tubulin* was used as an internal control [26] (Table 1).

### 2.8. Statistical Analysis

The experimental results were analyzed using SPSS 22.0 (IBM, Armonk, NY, USA), and the data are expressed as mean ± SD. One-way ANOVA was used for multiple-group comparison analysis, while two-tailed student’s *t*-test was used for two-group comparison analysis. Differences were considered significant at *p* < 0.05 or *p* < 0.01.

## 3. Results

### 3.1. Identification and Characteristics of Caprine circ_003628

**Figure 1:**
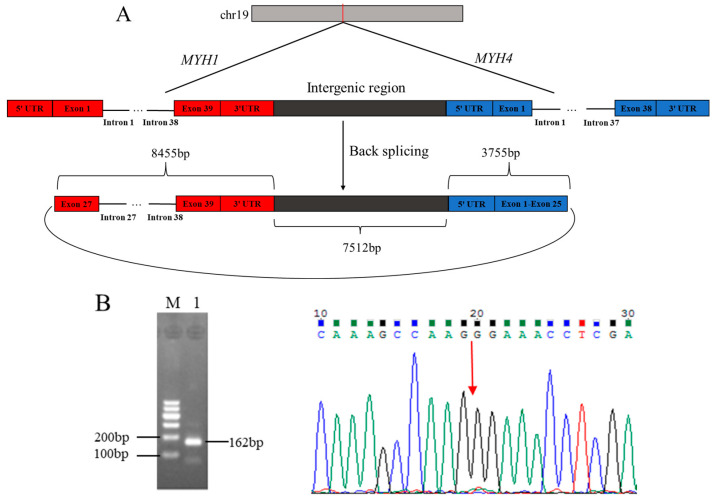
The structure of caprine circ_003628 derived from *MYH1* and *MYH4* (**A**) and validation of the authenticity of the circRNA using RT-PCR and Sanger sequencing (**B**). M: Marker; 1: circ_003628. The head-to-tail splice junction site for circ_003628 is marked with a red arrow.

The comparison of sequences from RNA-Seq data with Caprine Genome Assembly ARS1 revealed that circ_003628 was partially originated from 13 exons (exon 27–exon 39), 12 introns (intron 27–intron 38), and 3′-untranslated regions (UTR) of caprine Myosin Heavy Chain 1 (*MYH1*), and 25 exons (exon 1–exon 25) and 5′ UTR of Myosin Heavy Chain 4 (*MYH4*), as well as intergenic sequences between the two genes (Figure 1A). This suggested that circ_003628 was an exon-intron type of circRNA. The circ_003628 was located on goat chromosome 19, with a full length of 19,722 bp. RT-PCR amplicon of the expected size derived from circ_003628 was obtained using divergent primers (Figure 1B). Sanger sequencing result found that the head-to-tail junction site of circ_003628 was located at the joint of exon 27 of *MYH1* and exon 25 of *MYH4* (Figure 1B), which was consistent with the RNA-Seq result (Figure 1A). These results confirmed the authenticity of circ_003628.

**Figure 2:**
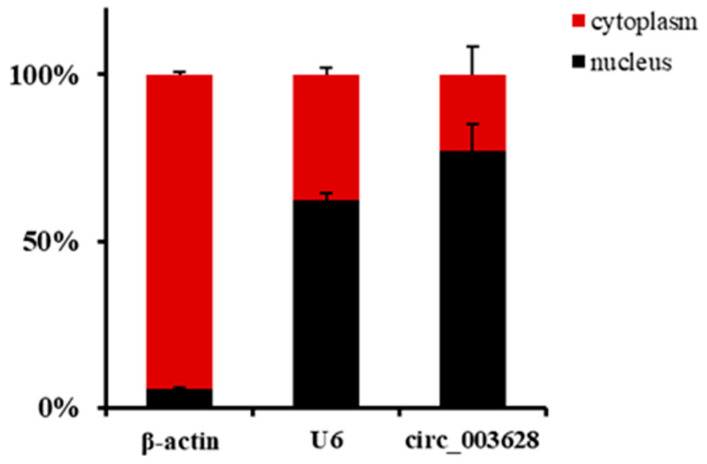
The localization of circ_003628, *β-actin* and *U6* in nuclei and cytoplasm of goat skeletal muscle satellite cells (SMSCs).

The RT-qPCR results in nuclei and cytoplasm of SMSCs showed that the expression levels of reference genes *U6* and *β-actin* in nuclei accounted for 62.42% and 5.54%, respectively. Accordingly, they were 37.58% and 94.46% in expression of cytoplasm, respectively. These results suggested that the nuclei and cytoplasm of SMSCs were successfully separated. A total of 22.93% circ_003628 were located in the cytoplasm of SMSCs, while 77.07% were expressed in the nuclei (Figure 2).

### 3.2. The Tissue Expression of circ_003628

**Figure 3:**
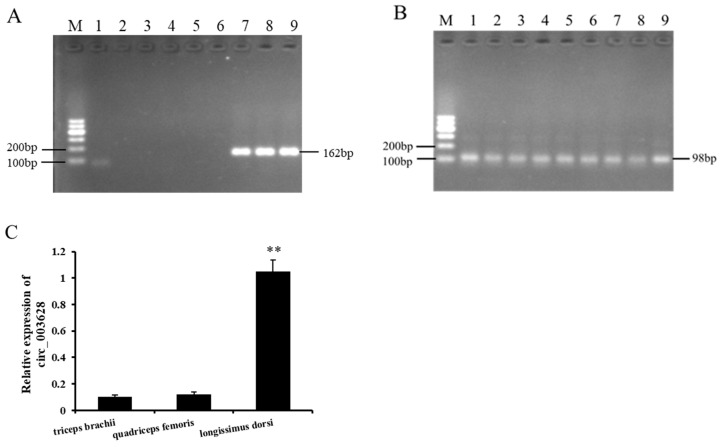
The expression of circ_003628 (**A**) and *GAPDH* (**B**) in different tissues collected from Ziwuling black goats. M: Marker; 1: heart; 2: liver; 3: spleen; 4: lung; 5: kidney; 6: testis; 7: *triceps brachii* muscle; 8: *quadriceps femoris* muscle; 9: *longissimus dorsi* muscle. (**C**) The relative expression level of circ_003628 in the three muscle tissues. ** *p* < 0.01.

The RT-PCR analysis of the nine different tissues collected from Ziwuling black goats showed that circ_003628 was only expressed in the *triceps brachii*, *quadriceps femoris* and *longissimus dorsi* muscle tissues, and its expression was not detected in the other six tissues (heart, liver, spleen, lung, kidney, and testis) (Figure 3A,B). The RT-qPCR results further revealed that circ_003628 had a higher level of expression in the *longissimus dorsi* than in the *triceps brachii* and *quadriceps femoris* (*p* < 0.01; Figure 3C).

### 3.3. The Expression Levels of circ_003628 and Its Parent Genes in Goat SMSCs

**Figure 4:**
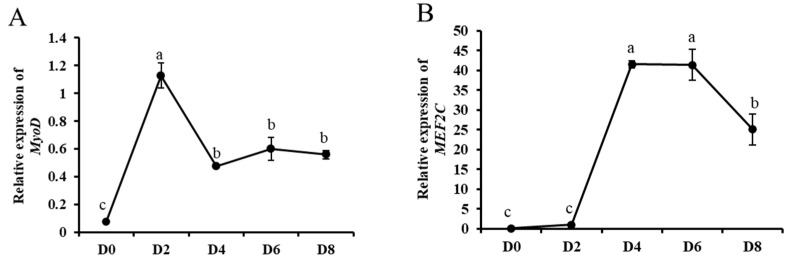

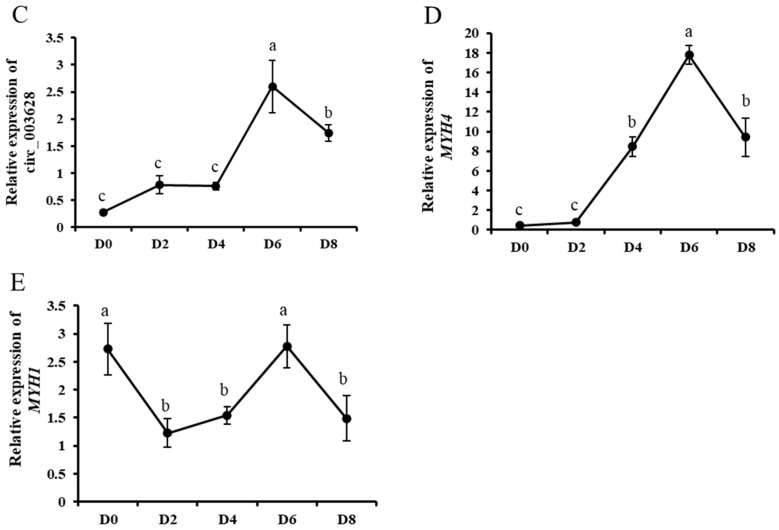
The expression levels of *MyoD* (**A**), *MEF2C* (**B**), circ_003628 (**C**), *MYH4* (**D**) and *MYH1* (**E**) on day 0, 2, 4, 6 and 8 after SMSCs differentiation. Different lowercase letters above the bars represent significant differences (*p* < 0.05).

In order to investigate the role of circ_003628 in differentiation of SMSCs, the expression levels of two myogenic differentiation marker genes *MyoD* and *MEF2C*, circ_003628 and its parent genes were detected during different differentiation periods of goat SMSCs. RT-qPCR results showed that the expression level of *MyoD* gradually increased from day 0 to day 2 of differentiation, and reached a maximum on day 2 after differentiation (Figure 4A), whereas the expression level of *MEF2C* increased significantly from day 2 to day 4 of differentiation and reached a maximum on day 4 after differentiation (Figure 4B). The results suggested that the induction differentiation of SMSCs was successful in this study. The expression levels of circ_003628 and *MYH4* increased gradually during differentiation and reached a maximum on day 6 after differentiation (Figure 4C,D). However, the expression level of *MYH1* had an ascent after an initial decline trend during goat SMSC differentiation until achieving a peak level on day 6 after differentiation (Figure 4E).

### 3.4. Circ_003628 Promotes Viability and Proliferation of SMSCs

**Figure 5:**
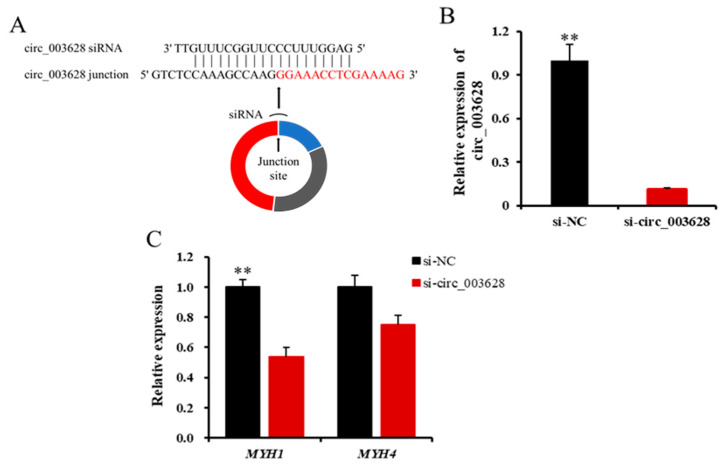
Schematic diagram of si-circ_003628 sequence specifically targeted the junction site of circ_003628 (**A**) and effect of si-circ_003628 on expression levels of circ_003628 (**B**) and its parent genes *MYH1* and *MYH4* (**C**) when si-circ_003628 was transfected into SMSCs. ** *p* < 0.01.

When si-circ_003628 structurally shown in Figure 5A was transfected into SMSCs for 48 h, it remarkably decreased the level of expression of circ_003628 compared with its NC group (*p* < 0.01, Figure 5B). This indicated that the caprine SMSCs was successfully transfected with si-circ_003628. Moreover, si-circ_003628 led to decreased expression of its parent genes *MYH1* (*p* < 0.01, Figure 5C) and *MYH4* (*p* = 0.053, Figure 5C).

**Figure 6:**
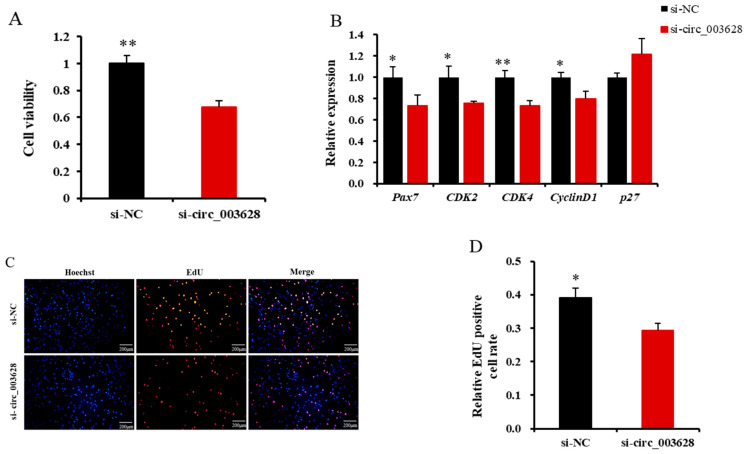
Effect of si-circ_003628 on viability and proliferation of goat skeletal muscle satellite cells (SMSCs). (**A**) Effect of si-circ_003628 on viability of SMSCs detected using CCK-8 assay. (**B**) The expression levels of cell proliferation marker genes *Pax7*, *CDK2*, *CDK4*, *CyclinD1* and *p27* when si-circ_003628 was transfected into SMSCs. (**C**) Effect of si-circ_003628 on proliferation of SMSCs detected using EdU assay. Scale bar indicates 200 µm. (**D**) The percentage of EdU-labeled positive SMSCs was calculated using ImageJ software. * *p* < 0.05 and ** *p* < 0.01.

The CCK-8 assay revealed that si-circ_003628 remarkably reduced the viability of SMSCs (*p* < 0.01, Figure 6A).

The RT-qPCR results revealed that si-circ_003628 significantly reduced the expression levels of *Pax7*, *CDK2*, *CDK4* and *CyclinD1* in SMSCs (*p* < 0.05, Figure 6B), while it promoted the expression level of *p27* (*p* = 0.108, Figure 6B).

The EdU assay found that the number of EdU-labeled positive SMSCs in the si-circ_003628 group was less than that in the si-NC group (Figure 6C,D). These results suggested that circ_003628 enhanced the viability of SMSCs as well as the proliferation of the cells.

### 3.5. Circ_003628 Promotes Differentiation of SMSCs

**Figure 7:**
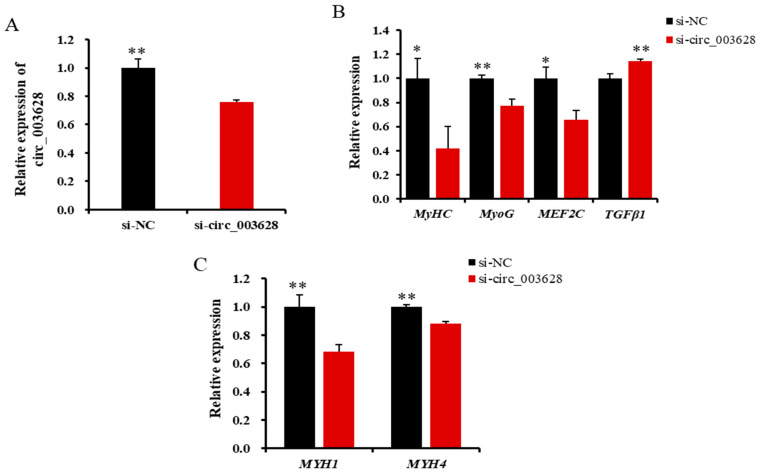
The relative expression level of circ_003628 (**A**), four cell differentiation marker genes (**B**) and its parent genes (**C**) when si-circ_003628 was transfected into SMSCs on day 6 after differentiation. * *p* < 0.05 and ** *p* < 0.01.

RT-qPCR results revealed that the expression level of circ_003628 in the si-circ_003628 group was significantly lower than in the si-NC group on day 6 after differentiation (*p* < 0.01, Figure 7A). This suggested that differentiated SMSCs was successfully transfected with si-circ_003628.

The interfering of circ_003628 significantly reduced the levels of expression of *MyHC*, *MyoG* and *MEF2C* (Figure 7B), while it increased the expression of *TGFβ1* (*p* < 0.01, Figure 7B). Moreover, the interfering of circ_003628 inhibited its two parent genes *MYH1* and *MYH4* (*p* < 0.01) in expression on day 6 after the initiation of differentiation (Figure 7C).

**Figure 8:**
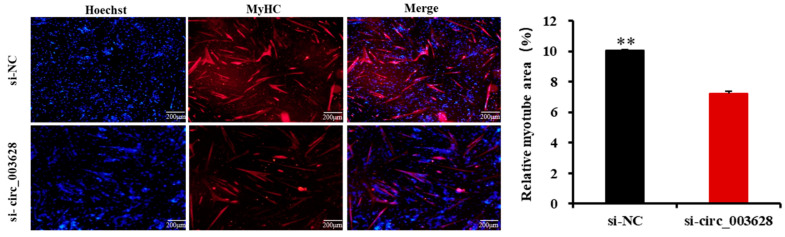
The effect of circ_003628 on area of MyHC-labeled positive myotubes detected using cell immunofluorescence. Scale bar indicates 200 µm. ** *p* < 0.01.

The immunofluorescence assay showed that si-circ_003628 also significantly decreased the area of MyHC-labeled positive myotubes (*p* < 0.01, Figure 8). These results demonstrated that circ_003628 promoted differentiation of SMSCs.

## 4. Discussion

This is the first study to report the origin, characteristics, and tissue expression of circ_003628, as well as its effect on goat SMSCs. In this study, the authenticity of circ_003628 in caprine muscle tissue was verified using RT-PCR and Sanger sequencing. These technologies have been widely used to affirm the authenticity of circRNAs, including circTTN [27], circFGFR4 [25], circ_015343 [28] and cricUBE2Q2 [29]. The sequence alignment results showed that circ_003628 was derived from caprine *MYH1* and *MYH4* genes. *MYH1* and *MYH4* belong to the sarcomeric MYH gene family that encodes different MyHC proteins [30]. *MYH1* encodes MyHC-IIX protein, expressed in intermediate muscle fibers (MyHC-IIX muscle fibers), while *MYH4* encodes MyHC-IIb protein, expressed in rapid-twitch glycolytic fibers (MyHC-IIb muscle fibers) [31,32]. It has been found that *MYH1* and *MYH4* may be related to the differentiation of SMSCs and muscle fiber hypertrophy. For example, Brown et al. [33] found that *MYH1* and *MYH4* were highly expressed in the later stages of C2C12 cells differentiation, suggesting that the two genes may affect the differentiation of the cells. An increase in the proportion of MyHC-IIb muscle fibers, as defined by MyHC-IIb protein, encoded by *MYH4*, was found to cause a hypertrophy of muscle fibers in pigs, thereby increasing meat production [34]. It was, therefore, inferred that circ_003628 produced by *MYH1* and *MYH4* may play important roles in the growth and development of caprine skeletal muscle tissues.

In this study, circ_003628 was only expressed in the three muscle tissues (*quadriceps femoris*, *triceps brachii*, and *longissimus dorsi*), but was not expressed in the other six tissues of goats. This likely reflects tissue-specific expression of circ_003628. A similar phenomenon was also observed by Wei et al. [35], who found that circLMO7, associated with muscle growth and development of Qinchuan cattle, was predominantly expressed in muscle tissue, but not expressed in intestine, spleen, lung, and kidney tissues. Interestingly, the expression level of circ_003628 was higher in the *longissimus dorsi* muscle than in the *triceps brachii* muscle and *quadriceps femoris* muscle. This may be due to the fact that the amount of movement of different muscles varies greatly, and the number of satellite cells in different parts may be different [36].

It was notable that circ_003628 was an exon-intron type of circRNA. The RT-qPCR results of circ_003628 using RNA extracted from goat SMSCs found that the circRNA was mainly located in nuclei. It has been reported that exon-intron circRNAs mainly located in nuclei can regulate the expression of their parent genes [37]. In this context, the effect of circ_003628 on the expression of the parent genes attracted our attention. In this study, the inhibition of circ_003628 decreased the expression of its parent genes *MYH1* and *MYH4* in both SMSCs and differentiated SMSCs. This result was similar to the finding reported by Li et al. [37], who found that circEIF3J and circPAIP2 promoted the expression of their parent genes *EIF3J* and *PAIP2* in Hela cells, respectively.

The proliferation and differentiation of SMSCs are crucial to the growth and development of animal skeletal muscle, as it has been proved that SMSCs are myogenic stem cells that can form myotubes through proliferation, differentiation and fusion after in vitro culture. In this context, SMSCs have been used as models for investigating the growth and development of animal skeletal muscle [38,39]. In order to study the effect of circ_003628 and its parent genes on differentiation of goat SMSCs, the expression levels of *MyoD*, *MEF2C*, circ_003628, *MYH1* and *MYH4* were detected in SMSCs during different differentiation stages. *MyoD* is considered to be an essential myogenic regulatory factor for SMSCs, which can make SMSCs begin to differentiate by activating the transcription of the other myogenic regulatory factors (MyoG, MEF2C and MyHC), but after the initiation of differentiation, its expression level gradually decreases [40]. Meanwhile, *MEF2C* is considered to be a differentiation marker gene and can promote the myogenic differentiation of SMSCs [41]. The expression levels of *MyoD* and *MEF2C* indicated that the induction differentiation of SMSCs was successful in the study. It was found that the expression levels of circ_003628 and *MYH4* were low in the early stage of differentiation. However, as the differentiation continued, their expression levels gradually increased and reached a peak on day 6 of differentiation. This suggested circ_003628 and *MYH4* may mainly function in the late stage of myogenic differentiation. The tendency of *MYH4* was similar to the finding reported by Brown et al. [33], who found that the expression levels of *MYH4* in C2C12 muscle cells significantly increased after differentiation and reached a maximum on day 8 of differentiation. The tendency of expression level may be related to the state of goat SMSCs during differentiation. The results suggest that circ_003628, *MYH1* and *MYH4* may affect the differentiation of SMSCs. Interestingly, the expression level of *MYH1* on day 0 of differentiation was higher than those on days 2, 4 and 8 of differentiation, indicating that *MYH1* may mainly affect SMSCs proliferation, but the specific mechanism remains to be further studied.

It was found that in the postnatal period of animals, increases of skeletal muscle mass mainly depend on the hypertrophy of muscle fibers, closely related to proliferation and differentiation of SMSCs [42,43,44,45]. In this study, si-circ_003628 decreased the expression levels of proliferation marker genes *Pax7*, *CDK2*, *CDK4* and *CyclinD1*. Meanwhile, si-circ_003628 increased the expression level of *p27*. It has been reported that *Pax7*, *CDK2*, *CDK4* and *CyclinD1* in expression promoted the proliferation of goat SMSCs, while *p27* inhibited the proliferation of goat SMSCs [27,46]. This taken together with the inhibited effect of si-circ_003628 on goat SMSCs, suggests that circ_003628 promotes proliferation of goat SMSCs by regulating the expression of the proliferation marker genes, eventually leading to the hypertrophy of muscle fibers and increase of skeletal muscle mass in goats.

It was also noteworthy that circ_003628 significantly increased the area of MyHC-labeled myotubes, as well as the expression of *MyHC*, *MyoG* and *MEF2C*, but it reduced *TGFβ1* in expression. The four genes have been reported to be associated with the differentiation of goat SMSCs. For example, the expression of *MyHC*, *MyoG* and *MEF2C* promoted the differentiation of goat SMSCs, while *TGFβ1* inhibited the differentiation of the cells [27,47,48]. Meanwhile, MyHC is a myogenesis marker protein, in that the area of MyHC-labeled myotubes is proportional to the degree of differentiation of SMSCs and the formation of myotubes [13,27]. These results suggest that circ_003628 promotes the differentiation of goat SMSCs, and further conclude that the circRNA may also increase caprine skeletal muscle mass by promoting differentiation of goat SMSCs.

## 5. Conclusions

This study verifies the authenticity of the presence, and describes tissue and timing-specific expression of circ_003628. The circRNA promotes the proliferation and differentiation of goat SMSCs. These results lay a theoretical foundation for elucidating the biological role of circ_003628 in caprine muscle growth and development.

## Figures and Tables

**Table 1:** Primer sequence information used for RT-qPCR.

Name *	Forward (5′ to 3′)	Reverse (5′ to 3′)	Amplicon Size (bp)
Circ_003628	GACTGTCTCCAAAGCCAAGG	CTGGTAGATGCCCACCTGAT	162
*MYH1*	AAGGGACTGTCCAGAGCAGA	CACAGAAGAGGCCCGAGTAG	225
*MYH4*	CACCCTGGAGGACCAACTGA	TTGCCTCGGGAAAGCTGAGAAA	165
*GAPDH*	ACACTGAGGACCAGGTTGTG	GACAAAGTGGTCGTTGAGGG	98
*U6*	GGAACGATACAGAGAAGATTAGC	TGGAACGCTTCACGAATTTGCG	68
*β-actin*	AGCCTTCCTTCCTGGGCATGGA	GGACAGCACCGTGTTGGCGTAA	113
*Pax7*	ACGAAGGGGACAAGAAGGAG	GGTAGTGGGTCCTCTCGAAG	213
*CDK2*	GACCAGCTCTTCCGGATCTT	ACAAGCTCCGTCCATCTTCA	160
*CDK4*	ACTTTGTGGCCCTCAAGAGT	CCTGAGGTCTTGGTCCACAT	215
*CyclinD1*	CGTCCATGCGGAAGATCGT	ACAGGAAGCGGTCCAGGTAGT	108
*p27*	CGGCGGTGCCTTTACTT	GCAGGTCGCTTCCTTATCC	127
*β-tubulin*	AGCGTATCTCAGAGCAGTTC	AATCCTCTTCCTCTTCTGCG	171
*MyHC*	CCACATCTTCTCCATCTCTG	GGTTCCTCCTTCTTCTTCTC	171
*TGFβ1*	CACGTGGAGCTGTACCAGAA	GCGAAAGCCCTCTATTTCCT	156
*MyoD*	GTGCAAACGCAAGACGACTA	GCTGGTTTGGGTTGCTAGAC	128
*MEF2C*	ATCCTGATGCAGACGATTCAG	GGTGGAACAGCACACAATCTT	115
*MyoG*	CGTGGGCGTGTAAGGTGT	GGCGCTCTATGTACTGGATGG	195

* The name of gene is indicated in italics.

## Data Availability

The data presented in this study are available on request from the corresponding author.
